# Clinical parameters predictive for sphincter-preserving surgery and prognostic outcome in patients with locally advanced low rectal cancer

**DOI:** 10.1186/s13014-020-01554-y

**Published:** 2020-05-06

**Authors:** Richard Partl, Marton Magyar, Eva Hassler, Tanja Langsenlehner, Karin Sigrid Kapp

**Affiliations:** 1 Department of Therapeutic Radiology and Oncology, Medical University of Graz, Comprehensive Cancer Center Graz (CCC), Auenbruggerplatz 32, 8036 Graz, Austria; 2 Division of Neuroradiology, Vascular and Interventional Radiology, Medical University of Graz, Comprehensive Cancer Center Graz (CCC), Auenbruggerplatz 9, 8036 Graz, Austria

**Keywords:** Predictive factors, Clinical parameters, Sphincter-preserving surgery, Low rectal cancer

## Abstract

**Background:**

Although controversial, there are data suggesting that clinical parameters can predict the probability of sphincter preserving procedures in rectal cancer. The purpose of this study was to investigate the association between clinical parameters and the sphincter-preserving surgery rate in patients who had undergone neoadjuvant combination therapy for advanced low rectal cancer.

**Methods:**

In this single center study, the charts of 540 patients with locally advanced rectal cancer who had been treated with induction chemotherapy-and/or neoadjuvant concomitant radiochemotherapy (nRCT) over an 11-year period were reviewed in order to identify patients with rectal cancer ≤6 cm from the anal verge, who had received the prescribed nRCT only. Univariate and multivariate analyses were used to identify pretreatment patient- and tumor associated parameters correlating with sphincter preservation. Survival rates were calculated using Kaplan-Meier analyses.

**Results:**

Two hundred eighty of the 540 patients met the selection criteria. Of the 280 patients included in the study, 158 (56.4%) underwent sphincter-preserving surgery. One hundred sixty-four of 280 patients (58.6%) had a downsizing of the primary tumor (ypT < cT) and 39 (23.8%) of these showed a complete histopathological response (ypT0 ypN0). In univariate analysis, age prior to treatment, Karnofsky performance status, clinical T-size, relative lymphocyte value, CRP value, and interval between nRCT and surgery, were significantly associated with sphincter-preserving surgery. In multivariate analysis, age (hazard ratio (HR) = 1.05, CI95%: 1.02–1.09, *p* = 0.003), relative lymphocyte value (HR = 0.94, CI95%: 0.89–0.99, *p* = 0.029), and interval between nRCT and surgery (HR = 2.39, CI95%: 1.17–4.88, *p* = 0.016) remained as independent predictive parameters.

**Conclusions:**

These clinical parameters can be considered in the prognostication of sphincter-preserving surgery in case of low rectal adenocarcinoma. More future research is required in this area.

## Background

According to the NCCN clinical practice guidelines combined-modality therapy consisting of surgery, concurrent fluoropyrimidine-based chemotherapy with ionizing radiation to the pelvis is recommended for patients with locally advanced rectal cancer stage II or III [1]. Using neoadjuvant rather than adjuvant RCT can lead to an improvement of local tumor control as well as the survival rate while at the same time reducing toxicity [2, 3]. The best treatment results are observed in patients with a good response to the neoadjuvant therapy. A histopathological complete response (ypCR) is associated with better disease-free survival (DFS) and overall survival (OS) [4]. There are also data indicating that a ypCR after nRCT is an independent indicator for the sphincter-preserving surgery rate [5].

There is a growing body of data that describe a relationship between tumor associated parameters and the tumor response. The most promising parameters are the tumor marker carcinoembryonic antigen (CEA), fibrinogen, genetic polymorphisms in epithelial growth factor receptor (EGFR) and thymidylate synthase (TS), bcl-2/bax and cyclooxygenase (COX)-2, clinical T-size, statin use, and the distance of the primary tumor from the anal verge [4, 6–13]. However, it remains unclear how these parameters can be used to predict the tumor response in clinical routine. What is also uncertain is whether pretherapeutic parameters can be used to predict the probability of sphincter-preserving surgery.

The medical decision on the type of operation to be performed has major implications for the patient’s future quality of life. The aim of this retrospective study was to identify patient- and tumor-associated parameters affecting the rate of sphincter-preserving surgeries. This additional information could enable surgeons to give patients better advice as to the probability of a sphincter-preserving surgery.

## Methods

The medical charts of 540 consecutive patients, who were referred for nCRT in the period 2004–2015 with histologically verified, locally advanced rectal carcinoma, were reviewed.

Patients with induction chemotherapy (*n* = 30), premature termination of radiotherapy (*n* = 1) and those who did not undergo surgery (*n* = 4) were excluded. Of the remaining 505 patients who received nCRT only, 280 had a tumor localization ≤6 cm from the anal verge. Only these patients were evaluated in the present study.

The pretherapeutic tumor stage was diagnosed by means of colonoscopy, rigid proctoscopy, digital rectal examination, endorectal ultrasound and pelvic CT/MRI. In order to rule out distant metastases, a thoracic and abdominal CT was performed. In view of the insufficient accuracy of current imaging methods, it was decided not to include the clinical lymph node staging in the evaluation.

The fluoropyrimidine-based concomitant chemotherapy consisted either of a continuous intravenous infusion with 5-fluoruracil (1000 mg/m2) administered during the first and last week of radiotherapy or an oral dose of capecitabine (1700 mg/daily) on each day of radiation treatment. The radiotherapy was planned and administered in a consistent manner throughout the study period. To exclude the small bowel, an open tabletop device (belly-board) was used when positioning the patient for the planning CT, and the perineum was marked with a radiopaque marker. Before CT, all patients received an oral contrast agent to visualize the small bowel. Radiotherapy was done with photon energies of 6 or 8 MEV in a 3D-conformal 3 or 4-field technique up to a total dose of 45–46 Gy in 23–25 fractions of 1.8 or 2 Gy/5 days weekly and was prescribed to the 95% isodose. After an interval of several weeks, either a total mesorectal excision (TME) or an abdominoperineal rectum resection (APR) was performed.

The following patient- and tumor-associated parameters, which were documented before the beginning of nRCT, were used in the analysis: patient age at the time of nCRT, gender, smoking, Karnofsky performance status (KPS), body mass index (BMI), clinical T-size (cT), histopathological subtype, histopathological tumor grading, full blood profile (erythrocytes, leukocytes, hemoglobin, thrombocytes, neutrophils, granulocytes, lymphocytes, serum lactate dehydrogenase (LDH), C-reactive protein (CRP), fibrinogen and the tumor markers CEA and CA19.9 (carbohydrate-antigen 19.9). Treatment-associated parameters analyzed included the histopathological tumor response and the interval between completion of nRTC and surgery. In addition, DFS and OS were calculated according to the type of surgery performed.

### Statistical analyses

Data are presented as mean and standard deviation or median and range for continuous data and absolute and relative frequency for categorical data. Potential predictors for sphincter-preserving surgery were analyzed using univariate logistic regression analysis. All variables that showed a *P* value of ≤0.05 in the univariate procedure were included in a stepwise multivariate logistic regression analysis. In addition, OS was analyzed from the date of surgery until death and DFS from date of surgery until local or distant recurrence or death using the log rank test. Furthermore Kaplan-Meier curves are given and 3 and 5 years survival rates were calculated. A *P* value of less than 0.05 was considered significant.

## Results

The median age of all 540 patients was 66.1 years (range: 32.1–88.4). Of the 280 patients selected for this analysis (with a tumor location ≤6 cm from the anal verge) the median age was 66.5 years (range: 32.2–88.4). A TME was performed in 158/280 patients (56.4%). Of these, 86.1% were staged as cT3 and 8.9% as cT4. In the remaining 122 patients (43.6%), in whom an APR was performed, 73% were staged as cT3 and 20.5% as cT4. The probability of undergoing sphincter-preserving surgery was 60.4% in cT3 and 35.9% in cT4.

Tumor downsizing (ypT < cT) was found in 164/280 patients (58.6%) in the specimens analyzed following surgery. Of these, 39 (23.8%) patients had a ypCR (ypT0 ypN0).

### Pretreatment parameters associated with sphincter-preserving surgery

Univariate analyses revealed significant associations of patient age, KPS (≤90% vs. 100%), clinical T- size (cT1–2 vs. cT3 vs. cT4), the erythrocyte value (< 4.5 vs. ≥4.5), relative lymphocyte value, CRP value and CRP reference values (≤8 vs. > 8) with the sphincter-preserving surgery rate. Pretreatment parameters and results of univariate analysis are given in Table 1.

**Table 1:** Pretreatment parameters: Results of univariate analysis

Parameter	n (missing%)	Sphincter preservation, n (%) or mean value ± SD	Abdominoperineal resection, n (%) or mean value ± SD	***p***-value
Overall	280 (0%)	158 (56.4%)	122 (43.6%)	
Mean age, years ± SD	280 (0%)	63.6 ± 11.6	67.4 ± 10.5	**0.006**
Gender	280 (0%)			0.096
Male		99 (62.7%)	88 (72.1%)	
Female		59 (37.3%)	34 (27.9%)	
Smoking	237 (15.4%)			0.640
Yes		27 (19.6%)	17 (17.2%)	
No		111 (80.4%)	82 (82.8%)	
Karnofsky performance status	164 (41.4%)			**0.004**
100%		76 (86.4%)	51 (67.1%)	
≤ 90%		12 (13.6%)	25 (32.9%)	
Body mass index (mean ± SD)	231 (17.5%)	26.0 ± 4.6	26.4 ± 4.1	0.484
Clinical T- size	280 (0%)			**0.018**
1–2		8 (5.1%)	8 (6.6%)	
3		136 (86.1%)	89 (73.0%)	
4		14 (8.9%)	25 (20.5%)	
Histopathological subtype	280 (0%)			0.052
Adenocarcinoma		151 (95.6%)	109 (89.3%)	
Andenocarcinoma (mucinous)		7 (4.4%)	13 (10.7%)	
Histopathological tumor grading	280 (0%)			0.295
1		10 (6.3%)	6 (4.9%)	
2		140 (88.6%)	104 (85.2%)	
3		8 (5.1%)	12 (9.8%)	
Erythrocyte count (T/l)	274 (2.1%)	4.7 ± 47	4.6 ± 86	0.764
Erythrocyte value (groups)	274 (2.1%)			**0.015**
below normal range (< 4.5)		34 (21.9%)	42 (35.3%)	
normal/above range (≥4.5)		121 (78.1%)	77 (64.7%)	
Leucocyte count (G/l)	275 (1.8%)	7.8 ± 6.6	7.7 ± 2.4	0.853
Leucocyte value (groups)	275 (1.8%)			0.412
normal range (≤11.3)		148 (95.5%)	113 (93.4%)	
above normal range (> 11.3)		7 (4.5%)	8 (6.6%)	
Hemogobin value (g/dl)	274 (2.1%)	13.5 ± 1.8	13.2 ± 2.0	0.207
Hemogobin value (groups)	274 (2.1%)			0.154
below normal range (< 13)		38 (24.7%)	39 (32.5%)	
normal range (13–17.5)		116 (75.3%)	81 (67.5%)	
Thrombocyte count (G/l)	271 (3.2%)	278 ± 97	284 ± 97	0.601
Thrombocyte value (groups)	271 (3.2%)			0.399
below normal range (< 140)		142 (93.4%)	114 (95.8%)	
normal range (140–440)		10 (6.6%)	5 (4.2%)	
Absolute neutrophil value (G/l)	243 (13.2%)	4.9 ± 1.63	5.28 ± 2.09	0.078
Relative neutrophile value (%)	230 (17.6%)	66.3 ± 9.0	68.0 ± 7.8	0.092
Absolute lymphocyte value (G/l)	265 (5.3%)	1.63 ± 0.52	1.56 ± 0.54	0.272
Absolute lymphocte value (groups)	265 (5.3%)			0.835
below normal range (< 1)		13 (8.6%)	9 (7.9%)	
normal range (1–4.8)		138 (91.4%)	105 (92.1%)	
Relative lymphocyte value (%)	273 (2.5%)	23.32 ± 7.40	21.24 ± 6.98	**0.021**
Relative lymphocyte value (groups)	268 (4.3%)			0.199
below normal range		50 (32.9%)	47 (40.5%)	
normal range		102 (67.1%)	69 (59.5%)	
LDH value (U/l)	259 (7.5%)	190 ± 70	186 ± 52	0.793
LDH value (groups)				0.850
normal range (≤240)		130 (88.4%)	98 (87.5%)	
above normal range (> 240)		17 (11.6%)	14 (12.5%)	
CRP value (mg/l)	255 (8.9%)	6.2 ± 13.9	10.6 ± 22.8	**0.003**
CRP value (groups)	255 (8.9%)			**0.008**
normal range (≤8)		124 (85.5%)	79 (71.8%)	
above normal range (> 8)		21 (14.5%)	31 (28.2%)	
CEA value (ng/ml)	200 (28.6%)	6.94 ± 12.60	15.75 ± 78.96	0.339
CEA value (groups)	200 (28.6%)			0.601
normal range (≤5)		75 (67.6%)	57 (64.0%)	
above normal range (> 5)		36 (32.4%)	32 (36.0%)	
CA 19.9 value (U/ml)	187 (33.2%)	38.94 ± 126.57	18.06 ± 27.99	0.780
CA 19.9 value (groups)	187 (33.2%)			0.453
normal range (≤37)		91 (86.7%)	74 (90.2%)	
above normal range (> 37)		14 (13.3%)	8 (9.8%)	

*Abbreviations*: *SD* Standard deviation, *T-size* Tumor-size according to the TNM classification, *LDH* Lactate dehydrogenase, *CRP* C-reactive protein, *CEA* Carcinoembryonic antigen, *CA19.9* Carbohydrate-antigen 19.9

### Treatment parameters associated with sphincter-preserving surgery

The univariate analysis showed a significant influence of the length of the interval between nRCT and surgery (≤6 weeks vs. > 6 weeks) on the rate of sphincter-preserving surgery. This was 56.7% for an interval < 6 weeks and 43.3% for an interval of > 6 weeks. No influence of ypCR (*p* = 0.73) or a reduction of the initial T-size was observed (0.72). Treatment-associated parameters and results of univariate analysis are shown in Table 2.

**Table 2:** Treatment parameters: Results of univariate analysis

Parameter	n (missing%)	Sphincter preservation, n (%) or mean value ± SD	Abdominoperineal resection, n (%) or mean value ± SD	***p***-value
ypCR	280 (0%)			0.730
Yes		23 (14.6%)	16 (13.1%)	
No		135 (85.4%)	106 (86.9%)	
T-downsizing	280 (0%)			0.721
Yes		94 (59.5%)	70 (57.4%)	
No		64 (40.5%)	52 (42.6%)	
Interval nRCT-Surgery, days	278 (0.7%)	50.2 ± 61.1	62.5 ± 157.4	0.420
Interval nRCT-Surgery (groups)	278 (0.7%)			**0.005**
< 6 weeks		89 (56.7%)	48 (39.7%)	
> 6 weeks		68 (43.3%)	73 (60.3%)	

*Abbreviation*: *ypCR* histopathological complete response to neoadjuvant therapy, *T-downsizing* Tumor-downsizing according to the TNM classification, *nRCT* neoadjuvant radiochemotherapy

**Table 3:** Parameters predictive for sphincter preservation in multivariate analysis

Parameter	OR	95% CI	***p***-value
Age, years	1.05	1.02–4.88	0.003
Relative lymphocyte value (%)	0.94	0.89–0.99	0.029
Interval nRCT-Surgery, weeks (< 6 vs. > 6)	2.39	1.17–4.88	0.016

*Abbreviation*: *nRCT* neoadjuvant radiochemotherapy, *OR* Odds ratio, *CI* Confidence interval

### Multivariate analysis of clinical and pathohistological parameters

The multivariate analysis was applied to all the parameters identified as relevant in the univariate analysis. Since there were strong correlations between the lymphocyte and neutrophil values and between the CRP and hemoglobin values, only the lymphocyte values and the CRP were considered in the further analyses. Three parameters were identified as independent significant parameters in the logistic regression analyses for sphincter preservation. These were the relative lymphocyte value (hazard ratio (HR) = 0.94, CI95%: 0.89–0.99, *p* = 0.029), age at time of irradiation (HR = 1.05, CI95%: 1.02–1.09, *p* = 0.003) and the interval between nRCT and surgery (HR = 2.39, CI95%: 1.17–4.88, *p* = 0.016). Results of multivariate analysis are given in Table 3.

### Influence of type of surgical treatment on survival

**Fig. 1:**
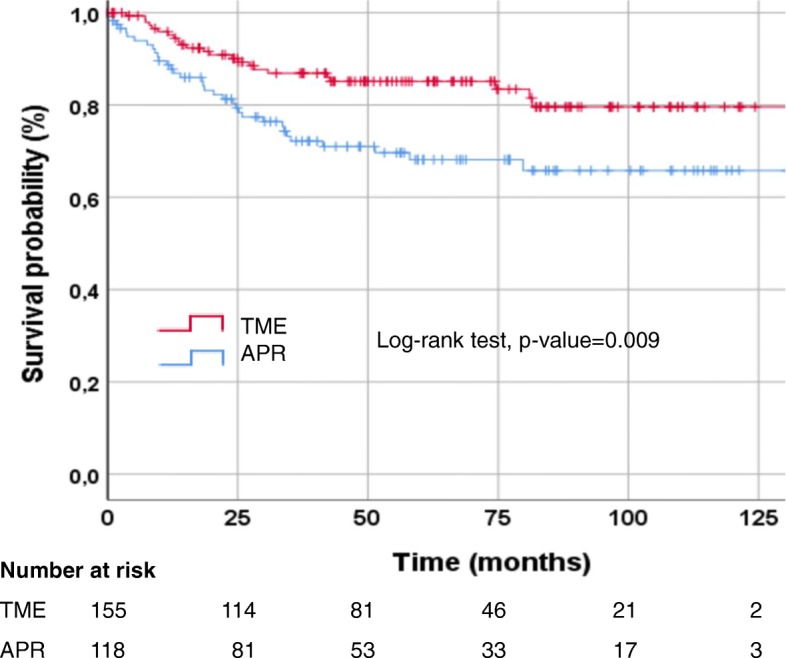
Kaplan Meier curves for disease-free survival by type of surgical treatment. Abbreviations: APR = abdominoperineal rectum resection; TME = total mesorectal excision

The median follow-up time of the 280 patients analyzed was 60.5 months (range: 0.0–159; mean: 66.4). In univariate analysis significant longer DFS (*p* = 0.009) and OS (*p* = 0.004) were observed in the sphincter-preserving surgery group compared to patients who had undergone APR. Kaplan-Meier estimates of DFS rates at 3 and 5 years were 86.9 and 85.1% for sphincter preserving surgery compared to 72.1 and 68.1%, for APR, respectively (Fig. 1).

**Fig. 2:**
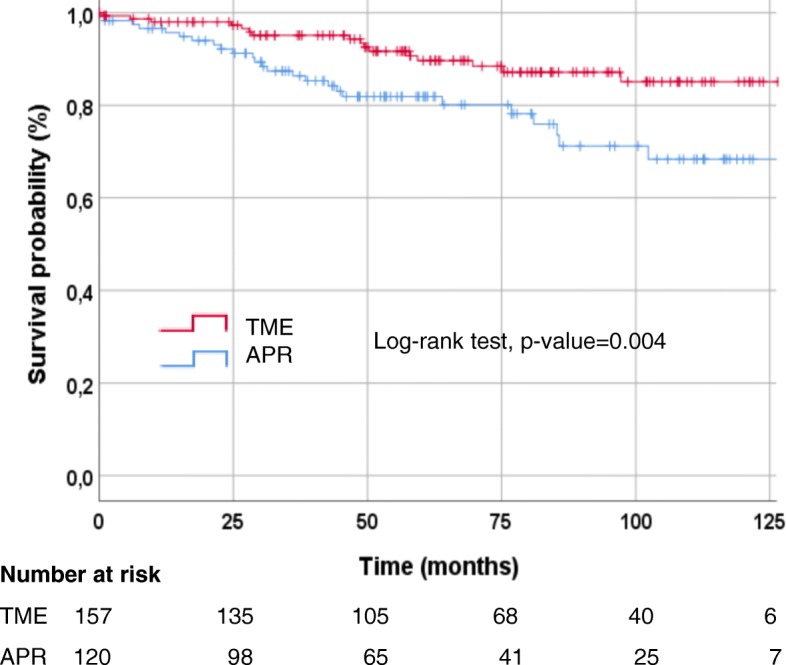
Kaplan Meier curves for overall-survival by type of surgical treatment. Abbreviations: APR = abdominoperineal rectum resection; TME = total mesorectal excision

Estimated OS rates at 3 and 5 years were 95.1 and 89.7% for sphincter preserving surgery and 86.4 and 81.9% for APR, respectively (Fig. 2).

## Discussion

Approximately 40% of the patients show no or only a small treatment response to nRCT [14]. In previous studies, absence of a tumor response was an independent adverse predictor of local tumor control and OS [4]. Opinions differ as to whether an nRCT has an effect on sphincter-preserving surgery rate. Reviews and metaanalyses found no significant difference in the APR rate between nRT with subsequent surgery and primary surgery, nor between nRT and nRCT [15, 16]. On the other hand, individual studies have shown that a good response to nRCT in patients with distal tumor localization influenced the rate of sphincter-preserving surgeries. According to Crane et al., patients with distal tumor location within ≤3 cm of the anal verge were given sphincter-preserving surgery more frequently if they had a clinical complete response (cCR) following nRCT [5]. The probability of patients with cCR of having a sphincter-preserving procedure was twice as high as those without cCR (44% vs. 22%; *p* = 0.01). In this way the authors were able to indirectly show an effect of nRCT on the chance of sphincter preservation. Response to nRCT did not affect surgical procedure in our sample. We presume this could be at first attributed to the fact observed by Crane et al., that in low rectal cancer patients with a distance > 3 cm to the anal sphincter who had pCR did not show higher sphincter-preserving rates. Additional evidence that nRCT does not improve sphincter-preservation is provided by a systemic review of randomised trials [17].

The distance of the tumor to the anal verge is an important parameter predictive for sphincter-preserving surgery, and this has been extensively studied [5, 18–22]. Tumors staged as T2–3, N0 in which a distal bowel clearance > 1 cm does not involve a major part of the external anal sphincter, a TME and intersphincteric distal dissection with hand-sewn colo-anal anastomosis is recommended [23–25]. An intersphincteric resection for ultra-low rectal cancer is associated with low morbidity, local recurrence rate of approximately 7%, disease-free survival of 78% and acceptable functional results [26]. According to the NCCN guidelines an APR should be performed when the tumor directly involves the anal sphincter or levator muscles or in cases where a margin-negative resection of the tumor would result in loss of anal sphincter function and incontinence [1]. Since the tumor localization is an independent predictive parameter for the probability of a sphincter-preserving operation, we limited our evaluation to patients with low rectal cancer. In this localization the available data on other predictive parameters is still very sparse. Apart from Crane et al., our analysis is the only one to date that has investigated exclusively patients with low rectal cancer treated with nRCT with a view to finding additional predictive factors for this decision.

The results of our analysis showed that the ability to perform sphincter-preserving surgery were significantly correlated with patient age, pretreatment relative lymphocyte value, and the interval between nRTC and surgery.

In our patient sample, younger patients were given sphincter-preserving surgery more often than older patients, which supports the findings of Temple et al., who also identified younger age as an independent factor for sphincter preservation [21]. In a study by Sun et al. [18] with 330 patients, the patient age only showed an effect on sphincter preservation in the univariate analysis; but younger age was a negative factor for sphincter preservation. A possible explanation is that younger patients might be diagnosed at a more advanced tumor stage. However, in our analysis there was no significant age difference between the groups with different tumor stages, so that we can rule out such an effect for our sample.

There is growing evidence that systemic immunity plays an important role in the tumor response to nRCT. Lymphocytes significantly contribute in cancer immune-surveillance, which inhibits tumor cell proliferation and metastasization (Ownby et al., 1983), in addition, elevated lymphocyte counts have been associated with improved prognosis in patients with different cancer entities [27]. The tumor-infiltrating lymphocyte density and the number of lymphocytes circulating in the peripheral blood have been shown to correlate strongly with tumor response rates [28]. High lymphocyte counts in the peripheral blood were significantly associated with better tumor response after nRCT in locally advanced rectal cancer [29, 30]. Also, a higher lymphocyte count nadir during nRCT was associated with higher ypCR rates and improved survival outcomes [31]. Our study confirms that the sphincter-preserving surgery rate in distal rectal carcinoma is influenced by the pretreatment lymphocyte count in peripheral blood (23.3 vs. 21.2; *p* = 0.021) but we could not confirm that the baseline lymphocyte count was associated with tumor response. Supporting our data, a Chinese group published that the absolute number of lymphocyte before and after therapy had no correlation with tumor response in two cohorts of overall 371 patients [32]. However, in the present study, we are unable to provide a clear explanation for the association between the pretreatment lymphocyte count and the sphincter-preservation rate. In that context, further studies to elucidate the role of additional potentially confounding factors such as tumor distance to the anal verge and surgeon’s expertise are warranted.

The effect of the length of time between completion of nRCT and surgery remains rather unclear. Tuchinsky et al. [33] showed that increasing this interval to > 7 weeks resulted in a significantly higher rate of ypCR and near-complete pCR of 35 and 17% (*p* = 0.03) respectively. In the group > 7 weeks, the DSF was also slightly longer (*p* = 0.05). Equally, Cotte et al. [34] observed an improvement of the pCR rate when the interval was extended to 6–8 weeks. However, the data on the nRCT-surgery interval are controversial in two respects. Firstly, although a longer interval increased the tumor response, and a good tumor response is assumed to be associated with a higher rate of sphincter-preserving surgery and improved OS, a longer nRCT-surgery interval did not improve these results in their cohorts [34, 35]. But secondly, there are also signs that extending the interval may even reduce the OS and metastasis-free-survivals [36, 37]. In practice, there is a wide variation in the timing of surgery (4–12 weeks) due to the fact that the optimal interval between nRCT and surgery remains controversial. Our results show that the interval is an independent parameter for sphincter preservation. An interval of up to 6 weeks increases the probability of a sphincter-preserving approach (HR = 2.39, CI95%: 1.17–4.88, *p* = 0.016). At intervals > 6 weeks, APR was performed more often (63.3% vs. 39.7%, *p* = 0.005). It seems that patients without a treatment response did not benefit from an interval > 6 weeks and therefore a shorter interval could be beneficial for nonresponders. We presume that longer intervals may enhance repopulation in non responders. We think the ideal interval requires a balance between allowing sufficient time for tumor response but before repopulation.

Retrospective comparisons of outcome have demonstrated that patients treated with APR had worse local control and OS [38]. Similarly, in our sample the extent of resection (sphincter-preserving TME vs. APR) seems to represent a prognostic factor for DFS and OS. Whether these findings are attributed to the surgical procedure alone or to patient- and tumor-related parameters is currently unclear. However, our observation might be supported by results from a recent retrospective study of 3633 patients with T3–4 rectal cancer tumors included in 5 large European trials suggesting that there is an association between the APR procedure itself and the increased risks of recurrence and death [39].

In interpreting our study and previous studies, it is important to remember that the endpoint TNM downstaging’ is based on the comparison between the clinical TNM stage and the histopathological TNM stage. With currently available imaging methods, it is not possible to evaluate the clinical stage reliably. 3 This applies especially to the assessment of lymph node status. A recent study by Brouwer et al. [40] yielded sensitivity, specificity, and positive and negative predictive values of 38, 87, 56, 76% in 2178 rectal cancer patients without neoadjuvant short course radiotherapy (SCRT) and 56, 67, 47 and 75% in 3401 patients with SCRT, respectively. The known lack of accuracy in clinical lymph node staging adds uncertainty to any investigation of predictive parameters based on it. This is the reason why we decided to exclude pretherapeutic lymph node status from our analysis.

We are aware of the shortcomings of this study: First, due to the retrospective nature of the present study we cannot rule out unknown confounders. Hence, our results have to be regarded as preliminary. Validation of our data in additional prospective studies with enough statistical power is imperative before firm conclusions can be drawn. Second, the distance of the lower tumor margin to the anal verge is undoubtedly an important independent factor for the determination of sphincter-preserving procedure. According to Sun et al. rectal cancer with a distance of the tumor from the anal verge of > 5 cm was shown to have a significant higher rate of sphincter-preservation [13]. Due to conflicting results between pre-treatment CT, MRT and rigid proctoscopy we decided not to incorporate the distance to the anal verge exactly. Third, the number of included patients is still too limited to draw firm conclusions.

In multiple studies [7, 8, 13], the serum fibrinogen value proved to be a promising independent predictor of the therapeutic response and the prognosis after nRCT. Until now, we have not had any data to show whether this parameter also influences the sphincter-preserving surgery rate. In our analysis, the fibrinogen value was not associated with sphincter-preserving surgery, but the result is weakened by the fact that this information was not available for 68.9% of the patients. A further limitation of our study may be that in 41.4% of the patients, the KPS was not recorded in the charts as a score. It was decided not to convert verbal descriptions of the patients’ general condition into scores.

In addition to tumor location, age, pre-treatment tumor fixation, the surgeons experience is a relevant factor affecting the sphincter-preservation rate. According to several authors the sphincter-preservation rate does depend on the experience of the surgeon. In centers with special expertise in colorectal cancer high rates of sphincter-preserving could be observed [20]. Several studies have also suggested that hospital volume or surgeon caseload have an independent influence [41, 42].

The extent to which our study is comparable with other studies of predictive parameters in rectal cancer patients is limited due to different inclusion criteria and treatment regimens. However, apart from 35 of 540 patients, who were excluded due to induction chemotherapy, premature termination of radiotherapy or not undergoing surgery, all of the remaining 505 patients referred for nCRT had received a uniform regimen of concomitant chemotherapy and radiotherapy. Of these, the 280 that met the selection criteria represent, to the best of our knowledge, one of the largest single-center studies investigating parameters predictive for sphincter preservation.

## Conclusions

Contributing to the body of information so far available, our results revealed that the patient age, the relative lymphocyte value prior to nCRT, and the interval between completion of nCRT and surgery were independently associated with sphincter-preserving surgery in our consistently treated cohort of patients with advanced low rectal cancer. Based on these preliminary data, future prospective, well-powered clinical studies should be performed to validate our findings and rule out potential confounders. If confirmed by additional studies, our findings might provide clinicians additional information about the probability of a sphincter-preserving procedure in case of low rectal adenocarcinoma at the beginning of an oncologic treatment and contribute to the identification of patients who could benefit from a more aggressive treatment approach.

## Data Availability

The datasets used and/or analysed during the current study are available from the corresponding author on reasonable request.

## Abbreviations

nRCT: Neoadjuvant radiochemotherapy
ypCR: Histopathological complete response
DFS: Disease-free survival
OS: Overall survival
CEA: Carcinoembryonic antigen
EGFR: Epithelial growth factor receptor
TS: Thymidylate synthase
TME: Total mesorectal excision
KPS: Karnofsky performance status
BMI: Body mass index
cT: Clinical T-size
LDH: Lactate dehydrogenase
CRP: C-reactive protein
CA19.9: Carbohydrate-antigen 19.9
HR: Hazard ratio
